# Dynamic evolution characteristics and driving factors of tourism ecosystem health in China

**DOI:** 10.3389/fpubh.2023.1127980

**Published:** 2023-02-20

**Authors:** Fei Lu, Huaiguo Ren, Xinglong Zhai

**Affiliations:** ^1^College of Culture and Tourism, Weifang University, Weifang, China; ^2^Editorial Department of Journal, Weifang University, Weifang, China

**Keywords:** tourism ecosystem health, dynamic evolution, Markov chains, quantile regression, China

## Abstract

Tourism ecosystem health is key to high-quality tourism development. China is now promoting sustainable development and high-quality transformation and upgrading of regional tourism; thus, the research on tourism ecosystem health is of practical significance. Based on the DPSIR model, an evaluation index system of tourism ecosystem health in China was constructed. Then the entropy weight method, spatial autocorrelation analysis, Markov chain analysis, and quantile regression were used to explore the dynamic evolution characteristics and driving factors of tourism ecosystem health in China from 2011 to 2020. The following conclusions were drawn: (1) The tourism ecosystem health in China showed an M-shaped fluctuation process as a whole, with significant spatial correlation and spatial difference. (2) There was a “path-dependent” and “self-locking” effect on the type transfer of tourism ecosystem health, and the type transfer was mainly between adjacent types in successive transfers, with the probability of downward transfer higher than upward transfer, and the geospatial background played a significant role in its dynamic evolution process. (3) In provinces with low tourism ecosystem health type, the negative effect of technological innovation capacity was more significant, and the influence coefficient of the positive effect of tourism environmental regulation and information technology level was larger, while in provinces with high tourism ecosystem health type, the negative effect of tourism industry agglomeration was more significant, and the influence coefficient of the positive effect of tourism industry structure and tourism land-use scale was larger.

## 1. Introduction

The tourism business, like other industries, has a paradoxical connection with the ecological environment due to the dual industrial qualities of environmental dependence and resource consumption (1–3). With the rapid growth of tourism and the advent of “mass tourism”, health issues affecting the industry's ecosystem have gradually surfaced (4, 5). Phenomena like excessive resource consumption, ecosystem degradation, biodiversity loss, and rising environmental pollution limited the resilience of sustainable tourism development (6–8). It has become a paramount practical concern to find a solution to integrate the interaction between ecological protection and tourism development and sustain the health of the tourism ecosystem. Exploring sustainable tourism development models has emerged as the most crucial challenge in China's tourism development as one of the nations with the fastest global tourist growth rates (9, 10). The report of the 20th CPC National Congress and the 14th Five-Year Plan for Tourism Development both place “promoting harmonious coexistence between man and nature” at the top of the overall agenda under the new development concept of “innovation, coordination, green, openness, and sharing”, and tourism ecosystem health has also grown to be a significant research area of high-quality tourism development. The research on tourism ecosystem health, with a particular focus on spatial and temporal dynamic evolution as well as driving factors, is thus crucial to the improvement of sustainable development and the high-quality transformation and upgrading of regional tourism in China.

Rapport, a Canadian scholar, introduced the concept of ecosystem health—which connects human health, human activity, and ecosystem change—into the field of ecosystems in 1979 (11). Since tourism ecosystems are stable, dynamic, and sustainable, the term “tourism ecosystem health” refers to the capacity of a system to maintain its own structural and functional integrity in the face of disturbances, such as those caused by human tourism activities (12, 13). It is widely acknowledged that a healthy tourism ecosystem is one in which the ecosystems themselves function in an orderly manner, satisfy the material and ecological needs of visitors and locals (14–16), and are capable of self-regulation and stress resistance (17, 18). The study of tourism ecosystem health, which concentrated on coordinating man-land connections, proliferated and flourished vigorously in the 1990s with the explosion of tourism sustainable development research and the International Society for Ecosystem Health (19–24). Previous studies have investigated tourism ecosystem health from the perspectives of disciplines such as geography, management, tourism, environment, and ecology (13, 25), including conceptual connotations, measurement and evaluation, spatial and temporal evolution characteristics, coordination status, trend prediction, influencing factors, and optimization paths (13, 25–29). Regarding research scales, tourism ecosystem health has been calculated at regional, urban, scenic, lakes, nature reserves, wetlands, and islands scales (25, 28–34). For the index system and measurement methods, the academic circles have started from the definition and characteristics of tourism ecosystem health, constructed a tourism ecosystem health evaluation index system based on the pressure-state-response (PSR) framework model, the drive-pressure-state-impact-response (DPSIR) framework model, and the vigor-organization structure-restoring force-service function-community crowd health-education level (VORSH) framework model (25, 28, 29, 31, 33), and then used the least squares method, the entropy weight method the fuzzy mathematical method and the analytic hierarchy method (AHP) to quantitatively measure tourism ecosystem health (26, 28, 31, 33). In recent years, constant attention has been given to analyzing factors influencing tourism ecosystem health. Most studies have used the gray correlation fuzzy assessment model, the obstacle degree model, the geographic detector model, etcetera (13, 29, 30).

To sum up, the existing studies have comprehensively examined tourism ecosystem health. They have produced a variety of findings that may be utilized as references in this paper. However, there are still several issues that need to be explored. First, there was broad agreement that the PSR model, the DPSIR model, or the VORSH model could be used to construct a tourism ecosystem health evaluation index system, but the coverage of specific indicators needed to be expanded and strengthened. Second, most previous studies used the traditional panel data model, which conforms to the normal distribution conditional mean, to identify the factors influencing tourism ecosystem health. Consequently, the possibility of different influencing factors in different regions of the tourism ecosystem health level was ignored. In addition, given the higher strategic value of sustainable tourism development and high-quality transformation and upgrading, little attention has been given to the spatial correlation and dynamic transfer of tourism ecosystem health at the national scale in the results of the available study, which are mostly focused on the dimensions of specific areas and economic zones. This paper attempts to deepen the previous research further based on these three deficiencies. Therefore, we selected 30 provinces (excluding Tibet, Hong Kong, Macao, and Taiwan) in China as the research subjects, then constructed a tourism ecosystem health evaluation index system based on the DPSIR model and used the entropy method to assess that health from 2011 to 2020. Meanwhile, the dynamic evolution characteristics of the tourism ecosystem health were explored through the entropy weight method, spatial autocorrelation analysis, and Markov chain analysis. Moreover, the panel quantile regression model was used to identify the driving forces behind the shifting trends in tourism ecosystem health under various quantile conditions. The research findings of this paper are intended to comprehensively understand the dynamic evolution characteristics and driving factors of tourism ecosystem health in China and provide a theoretical basis and decision-making reference for provinces with different levels of tourism ecosystem health so that it can promote the coordinated development of tourism and the eco-environment.

## 2. Materials and methods

### 2.1. Entropy weight method

The subjective assignment approach introduces the impact of human factors. However, the entropy weighting method uses the original information of the indicators as the foundation for assigning weights, which can adequately reflect the significance of each indicator in the comprehensive index. The entropy weight method calculated the evaluation index weight of tourism ecosystem health. The following are the specific steps for implementation (35):

The first step is to standardize the evaluation indexes.


(1) $$\begin{array}{l} \text{Positive index}:{\text{x}}_{\text{ij}}^{{}^{\text{′}}}=\frac{{\text{x}}_{\text{ij}}-\min{\text{ x}}_{\text{ij}}}{\max{\text{ x}}_{\text{ij}}-\min{\text{ x}}_{\text{ij}}} \end{array}$$

(2) $$\begin{array}{l} \text{Negative index}:{\text{x}}_{\text{ij}}^{{}^{\text{′}}}=\frac{\max{\text{ x}}_{\text{ij}}-{\text{ x}}_{\text{ij}}}{\max{\text{ x}}_{\text{ij}}-\min{\text{ x}}_{\text{ij}}} \end{array}$$

In Formulas (1) and (2), *x*_*ij*_represents the original index *j* in the region *i*; *x*${}_{ij}^{{}^{\prime }}$represents the index after standardization processing; max*x*_*ij*_ and min*x*_*ij*_represent the maximum and the minimum values of index *j*, respectively.

The second step is to calculate the weight. In Formulas (3) and (4), *w*_*ij*_is the proportion of index *j* under the region *i*; *e*_*j*_ is the entropy value of index *j*, and ϕ_*j*_ is the index weight. The specific Formula is as follows:


(3) $$\begin{array}{lll} w_{ij} & = & \frac{x_{ij}^{{}^{\prime }}}{\overset{m}{\underset{i=1}{\sum }}x_{ij}^{{}^{\prime }}},\;e_{j}=-\frac{1}{\ln\;m}\overset{m}{\underset{i=1}{\sum }}w_{ij}\times \ln\;w_{ij} \end{array}$$

(4) $$\begin{array}{lll} \varphi_{j} & = & \frac{\left(1-e_{j}\right)}{\overset{m}{\underset{j=1}{\sum }}\left(1-e_{j}\right)} \end{array}$$

The third step is a comprehensive evaluation index of tourism ecosystem health (*TEH*_*i*_).


(5) $$\begin{array}{l} TEH_{i}=\overset{m}{\underset{j=1}{\sum }}\varphi_{j}\times w_{ij} \end{array}$$

Integrating the results of the actual measurement of tourism ecosystem health in China and the synthesis of existing studies (28, 33), the tourism ecosystem health of 30 provinces in China was divided into four types: non-healthy level, sub-healthy level, generally healthy level, and very healthy level (Table 1).

**Table 1:** Tourism ecosystem health level standard.

**Health state**	**Unhealthy level**	**Sub-healthy level**	**Generally healthy level**	**Very healthy level**
Health type	I	II	III	IV
Health value	(0,0.30]	(0.30,0.40]	(0.40,0.55]	(0.55,1]

### 2.2. Global spatial autocorrelation analysis

Global spatial autocorrelation analysis, frequently measured by the global Moran's I index, is used to evaluate the overall spatial dependency between different geographical areas (36). In this paper, the global Moran's I index can reflect the spatial autocorrelation of tourism ecosystem health in China as a whole. The Formula for calculating the global Moran's I index is:


(6) $$\begin{array}{l} Global\;Moran^{\prime }s\text{I}=\frac{n\overset{n}{\underset{i=1}{\sum }}\overset{n}{\underset{j=1}{\sum }}W_{ij}\left(x_{i}-\bar{x}\right)\left(x_{j}-\bar{x}\right)}{\overset{n}{\underset{i=1}{\sum }}\overset{n}{\underset{j=1}{\sum }}W_{ij}\overset{n}{\underset{i=1}{\sum }}{\left(x_{i}-\bar{x}\right)}^{2}} \end{array}$$

In Formula (8), *n* represents the total number of provincial samples, which is 30 here; *x*_*i*_and *x*_*j*_represent the tourism ecosystem health measures of provinces *i* and *j*, respectively; $\overset{\bar{}}{x}$ is the arithmetic means of the tourism ecosystem health of all provinces; *W*_*ij*_ is the adjacency weight matrix, which indicates the adjacency relationship between two provinces, and *W*_*ij*_= 1 when provinces *i* and *j* are adjacent, otherwise it is 0. The value of global Moran's I index is [−1, 1]. The more the value of this index tends to 1, the stronger the correlation in the tourism ecosystem health space. The value of this index tends to 0, which indicates spatial decorrelation and random distribution in space.

### 2.3. Markov chain analysis method

The Markov chain, a transition matrix and the simplest stochastic model has been extensively used for state change studies at various spatial scales (37). In this paper, the Markov chain analysis method was used to calculate the initial and transfer probability of different states of tourism ecosystem health, and the trend over time was determined. Assuming that *M*_*t*_ = [ *M*_1,*t*_*,M*_2,*t*_*,M*_3,*t*_*,… M*_*k,t*_] is a vector of 1 × *k* state probability distributions in year *t*, the transfer between various types of tourism ecosystem health in different years can be represented by a Markovian transfer probability matrix of order *z* × *z*:


(7) $$\begin{array}{l} P\text{=}\left(\begin{array}{ccc} p_{11} & \ldots & p_{1k}\\ \vdots & \ddots & \vdots \\ p_{k1} & \cdots & p_{kk} \end{array}\right) \end{array}$$

In Formula (9), *p*_*ij*_ represents the probability of a random process transitioning from state type *i* in year *t* to state type *j* in year *t* + 1, and it can be calculated as α_*ij*_/α_*i*_; α_*ij*_ represents the number of provinces of type *i* in year *t* transferred to type *j* in year *t* + 1 and α_*i*_ represents the number of provinces of type *i* in all years.

To examine the relationship between the probability of transfer of tourism ecosystem health types and neighboring provinces, the spatial Markov chain analysis incorporated the spatial lag factors into the analytical framework based on the traditional Markov chain analysis (38). The traditional z-order transition probability matrix (*z* × *z*) is broken and decomposed into *n* conditional transition probability matrices (*z* × *z* × *z*) by the spatial Markov chain transition probability matrix. In the z-th condition matrix, *P*_*zij*_ designates the probability that a province *a* that was in type *i* in year *t* transfers into type *j* in year t + 1, conditional on a spatial lag type of *z*. The spatial lag value for province *a* is a weighted average of the tourism ecosystem health of the province's spatial neighbors. The calculation Formula is:


(8) $$\begin{array}{l} Lag_{a}=\sum Y_{b}W_{ab} \end{array}$$

In Formula (10), *W*_*ab*_ is the adjacency weight matrix, which indicates the adjacency relationship between two provinces, and *W*_*ab*_ = 1 when provinces *a* and *b* are adjacent; otherwise, it is 0. *Y*_*b*_ represents the tourism ecosystem health in province *b*; *Lag*_*a*_ represents the spatial lag value of province *a*, indicating the state of the neighborhood of province a. By comparing the traditional Markov chain matrix and the spatial Markov chain matrix, it is possible to determine the importance of the surrounding area on the probability of a particular spatial province's type transition.

### 2.4. Panel quantile regression model

Koenker and Basett proposed the quantile regression model to overcome the shortcomings of the traditional regression method, which can only obtain the average influence of the explanatory variables on the dependent variable (39). The difference in significance levels and regression coefficients at various quantiles in the quantile regression model can reflect the heterogeneity of the influence of the explanatory variables on the explained variables. Tourism ecosystem health varies from province to province, and explanatory variables affect those with high tourism ecosystem health differently than those with low tourism ecosystem health. Therefore, the quantile regression model can comprehensively, systematically, and dynamically reveal the effect of driving factors on tourism ecosystem health. For the panel data, the quantile regression model is set as follows (40):


(9) $$\begin{array}{l} Y_{it}=x_{it}^{T}\beta_{i}+\alpha_{i}+\mu_{it},\;\\ \text{ (}i=1,2,\cdots K;\;t=i=1,2,\cdots T\text{)} \end{array}$$

(10) $$\begin{array}{lll} Q_{y_{it}}\left(\tau |x_{it},\alpha_{i}\right) & = & x_{it}^{T}\beta \left(\tau_{q}\right)+\alpha_{i} \end{array}$$

In Formula (11) and (12), *x*_*it*_ represents the independent variable of province *i* in the 1 × *k* dimension of year *t*; β_*i*_ and α_*i*_are the parameters to be estimated, and μ is a random error; *Q*_*yit*_ represents the tourism ecosystem health at the quantile under the given explanatory variable conditions; τ represents the quantile points. The following Formula generally calculates the parameter β:


(11) $$\begin{array}{l} \beta =\arg\min_{\alpha ,\beta }\overset{Q}{\underset{q=1}{\sum }}\overset{T}{\underset{t=1}{\sum }}\overset{N}{\underset{i=1}{\sum }}W_{k}\rho_{\tau }\left[y_{it}-x_{it}^{T}\beta \left(\tau_{q}\right)-\alpha_{i}\right] \end{array}$$

In Formula (13), ρ_τ_represents the quantile loss function; *W*_*k*_ represents the weight coefficient of the *k* quantile; β(τ_*q*_) represents the influence coefficient of the *k* quantile.

Based on the current state of China's tourism industry and data availability, six indicators were selected to quantify the factors driving the change process in the spatial and temporal patterns of tourism ecosystem health under multiple factors. These indicators include tourism industry structure, tourism industry agglomeration, tourism environmental regulation, information technology level, technological innovation capacity, and tourism land-use scale (Table 2).

**Table 2:** Variables and explanations of tourism ecosystem health influencing indicators.

**Driving factors**	**Abbreviation**	**Definition**
Tourism industry structure	TIS	Tourism industry structure rationalization index
Tourism industry agglomeration	TIA	Location quotient
Tourism environmental regulation	TER	Cost of pollution control/tourism revenue
Information technology level	INFO	Internet broadband access users
Technological innovation capacity	TIC	R&D expenditure
Tourism land-use scale	TLS	Tourism revenue/provincial area

### 2.5. Evaluation index system and data source

The PSR model served as the foundation for the DPSIR model, which was first proposed and utilized by the European Environment Agency (EEA) to provide a more thorough understanding of the interactions and feedback mechanisms between human activities and the biological environment (41). The DPSIR model for tourism ecosystem operations is as follows: for an extensive period, economic and social development and tourism demand act as driving forces (D) on the ecosystem, resulting in several pressures (P) on the environment and changing the health state (S) of the tourism ecosystem, which in turn has a variety of impacts (I) on individuals, nature, and society, forcing human society to respond to ecological changes (R). Combining the characteristics of tourism ecosystems and the concept of tourism ecosystem health, this paper constructs a set of indicators for assessing the tourism ecosystem health in China (13, 42), as shown in Table 3.

**Table 3:** Tourism ecosystem health evaluation index system.

**Dimension**	**Sub-dimension**	**Index (unit)**	**Positive/negative**
Driving force (D)	Economic development	Per capita GDP (yuan)	Positive
		Disposable income per resident (yuan)	Positive
	Social life	Natural population growth rate (%)	Negative
		Urbanization rate (%)	Negative
	Tourism demand	Growth rate of tourists (%)	Negative
Pressure (P)	Ecological environment	SO_2_ emission per unit area (t/hm^2^)	Negative
		Sewage discharge density (m^3^/hm^2^)	Negative
	Social life	Per capita daily water consumption (m^3^/person)	Negative
		Population density (person/hm^2^)	Negative
	Tourism reception	Tourist traffic pressure (person times/km^2^)	Negative
		Visitor density (person times/hm^2^)	Negative
State (S)	Ecological environment	Proportion of good air quality days (%)	Positive
		Percentage of forest cover (%)	Positive
		Per capita arable land (hectare/person)	Positive
	Tourism resources	Tourism resources density (units/million km^2^)	Negative
		Tourism resource taste (%)	Positive
	Tourism facilities	Density of star-rated hotels (units/million km^2^)	Negative
		Travel agency density (units/million km^2^)	Negative
	Tourism economy	Domestic tourism revenue (billion yuan)	Positive
		Tourism foreign exchange earnings (USD billion)	Positive
Impact (I)	Ecological environment	Decline rate of ecological land (%)	Negative
	Sudden environmental incidents	Rate of increase in sudden environmental incidents (%)	Negative
	Economic structure	Proportion of total tourism revenue in GDP (%)	Positive
		Proportion of tertiary industry in GDP (%)	Positive
Response (R)	Government regulation and control	Proportion of environmental investment in GDP (%)	Positive
		Number of college students per 100000 population (%)	Positive
	Environmental governance	Urban domestic sewage treatment rate (%)	Positive
		Harmless domestic waste treatment rate (%)	Positive

The data in this paper were mainly derived from the “China Statistical Yearbook”, “China Tourism Statistical Yearbook”, “China Culture and Tourism Yearbook”, the yearbooks of each province, and the Statistical Bulletin of National Economic and Social Development of each province. Some missing data was supplemented using the linear interpolation method. Following data collection, panel data for 30 Chinese provinces from 2011 to 2020 were obtained.

## 3. Results

### 3.1. Spatial and temporal characteristics of tourism ecosystem health

#### 3.1.1. Time-series evolutionary characteristics

**Figure 1:**
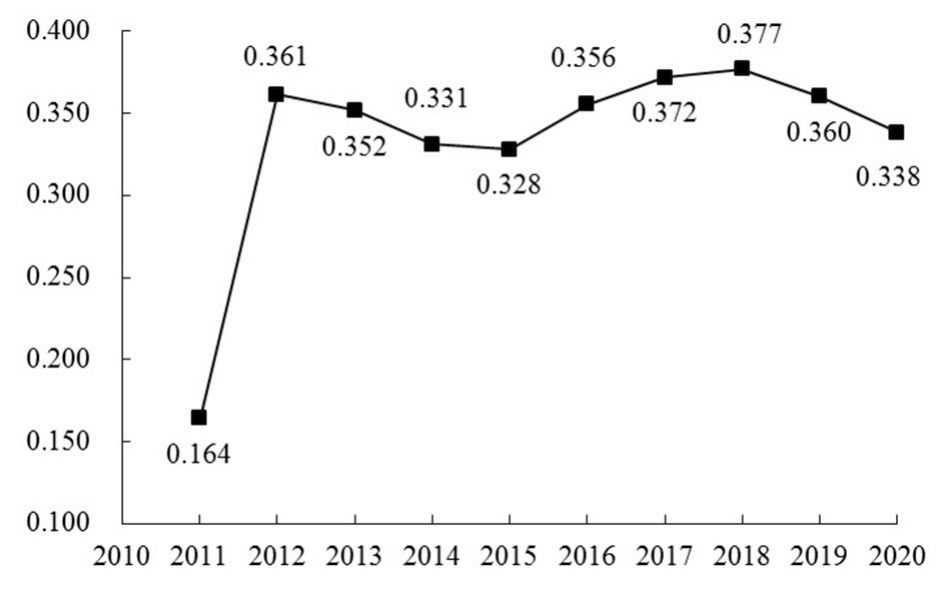
The trend of tourism ecosystem health in China from 2011 to 2020.

According to Formula (1)–(7), the tourism ecosystem health of 30 provinces in China from 2011 to 2020 was evaluated based on the constructed evaluation index system of tourism ecosystem health, and the trend of change was plotted (Figure 1). During the study period, the tourism ecosystem health in China showed an M-shaped fluctuation process of “increasing-decreasing-increasing-decreasing”, with a three-stage evolution. In the first stage (2011–2012), tourism ecosystem health rose from 0.164 in 2011 to 0.361 in 2012, an increase of 120.12%. China introduced the 12th Five-Year Plan in 2011, and the government has emphasized structural adjustment, energy conservation, emission reduction, and coordinated regional development. Moreover, the Opinions on Accelerating Tourism Development and the National Ecotourism Development Outline (2008–2015) have clearly defined initiatives to develop low-carbon tourism and green tourism, reinforcing the correlation between ecological protection and tourism development. Policy guidelines have promoted the green transformation and enhancement of China's tourism industry, resulting in a more substantial improvement in tourism ecosystem health. In the second stage (2012–2015), tourism ecosystem health declined slowly from 0.361 in 2012 to 0.328 in 2015, a decrease of 3.3%. The adverse effects of the crude growth of the tourism economy at this stage have gradually emerged. The intensification of resource and environmental constraints has diminished tourism ecosystem health. Tourism ecosystem health in the third stage (2015–2020) showed an inverted N-shaped trend. At the beginning of this stage, China's economy entered a period of new normal, with the deepening of the concept of ecological civilization and increased government efforts to combat environmental pollution, resulting in a rise in tourism ecosystem health. However, with the impact of the new crown epidemic (Covid-19) on both the supply and demand sides, tourism ecosystem health has gradually shifted toward a downward phase.

**Figure 2:**
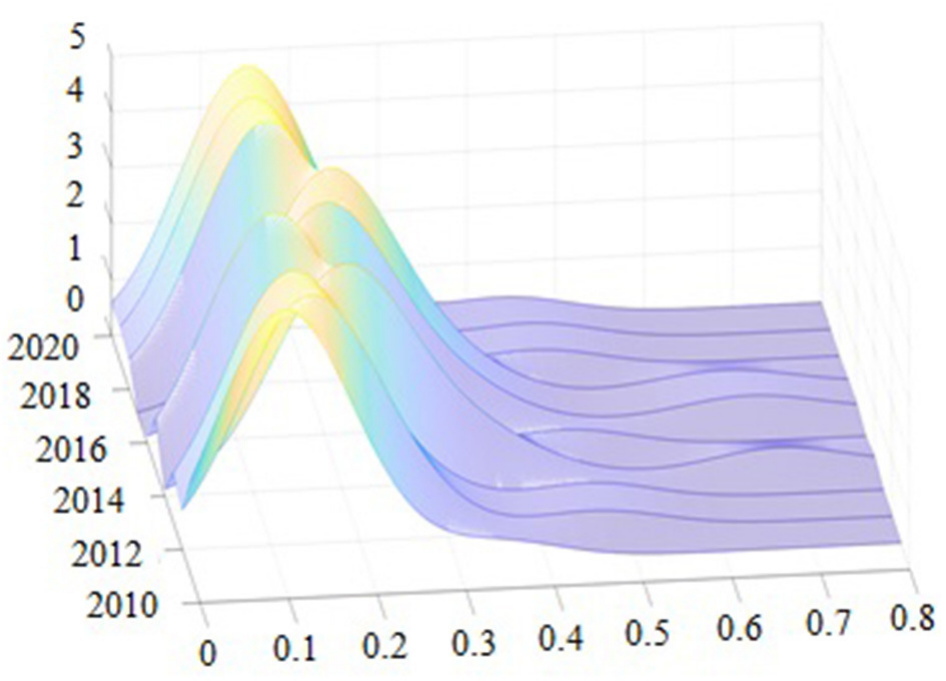
The Kernel density estimation of tourism ecosystem health in China.

The kernel density of the Gauss kernel function, depicted by Matlab R2011b software in Figure 2, was estimated to define the time-varying process of the absolute differences in the tourism ecosystem health across provinces. Then, utilizing the distribution location, distribution trend, and distribution extensibility, the kernel density was estimated using the Gauss kernel function to illustrate the dynamic evolution of the tourism ecosystem health in China. The nuclear density distribution curve shifted first to the right, then to the left, then to the right, and finally to the left from 2011 to 2020. This indicates that the tourism ecosystem health in China has undergone the process of “increasing-decreasing-increasing-decreasing”, which is in line with the trend analyzed above. The overall kernel density exhibited a right-trailing phenomenon concerning the extension of the distribution over time, indicating that the gap between China's tourism ecosystem health and the average has widened and that the types of provinces with high tourism ecosystem health levels were increasing faster while the types of provinces with low tourism ecosystem health levels were decreasing. From a morphological point of view, the overall kernel density curve did not show a decreasing peak or increasing width. However, the sample period was characterized by multiple peaks, implying a particular gradient of differences in the tourism ecosystem health in China.

#### 3.1.2. Spatial differentiation characteristics

According to Formula (8), combined with the measured value of tourism ecosystem health, using the Rook spatial weight matrix (Hainan and Guangdong were set as neighbors to avoid the “island phenomenon”), the spatial autocorrelation of tourism ecosystem health in China is tested and analyzed, and the global Moran's I index test results were calculated using Stata 16.0 software (Table 4). It can be seen from Table 4 that the global Moran's I index of tourism ecosystem health in China from 2011 to 2020 was all positive and passed the statistical test, reflecting a solid spatial correlation. China's spatial distribution of tourism ecosystem health was not isolated and random. However, it showed apparent spatial dependence and agglomeration, such as convergence between provinces with high tourism ecosystem health level types, forming a “high-high” agglomeration pattern, and the convergence between provinces with low tourism ecosystem health level types, forming a “low-low” agglomeration pattern. From 2012 to 2020, Moran's I index of tourism ecosystem health continued to decline in fluctuation, showing a trend of weakening spatial dependence, which, to a certain extent, indicated that China's tourism ecosystem health was gradually showing a coordinated and linked trend of regional integration.

**Table 4:** Global Moran's I index from 2011 to 2020.

**Year**	**2011**	**2012**	**2013**	**2014**	**2015**	**2016**	**2017**	**2018**	**2019**	**2020**
Moran's I	0.298	0.305	0.254	0.230	0.240	0.224	0.190	0.176	0.192	0.133
Z-scores	2.819	2.879	2.472	2.262	2.337	2.219	1.927	1.808	1.957	1.439
*P*-value	0.002	0.002	0.007	0.012	0.010	0.013	0.027	0.035	0.025	0.075

**Figure 3:**
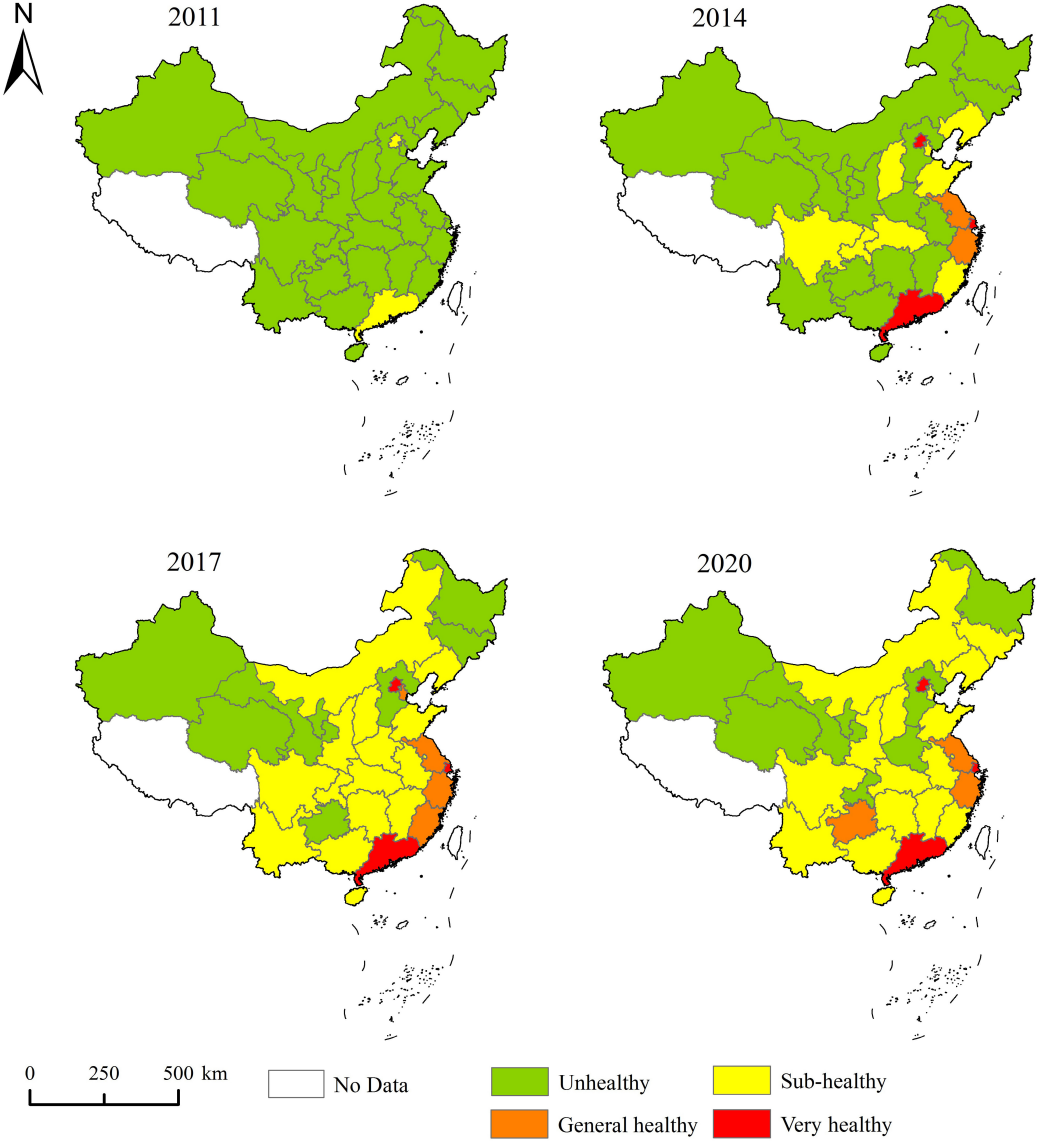
Spatial distribution patterns of tourism ecosystem health.

With the help of ArcGIS 10.2 software, the spatial distribution map of tourism ecosystem health in 2011, 2014, 2017, and 2020 was drawn, and the tourism ecosystem health was divided into four types, which were non-healthy level, sub-healthy level, generally healthy level and very healthy level from low to high (Figure 3). During the inspection period, the provinces with higher tourism ecosystem health levels were mainly concentrated in Beijing, Shanghai, Guangdong, Jiangsu, and Zhejiang, accounting for a small portion of the total. This showed significant regional differences in the tourism ecosystem health in China, which were out of balance. Specifically, 28 provinces with non-healthy tourism ecosystem health levels in 2011, except for Beijing and Guangdong. Compared with 2011, Jiangsu and Zhejiang joined the provinces with generally healthy tourism ecosystem health levels, while Liaoning, Shandong, Fujian, and other provinces withdrew from the non-healthy level. In 2017, the provinces of the very healthy level type remained unchanged. Tianjin and Fujian went up from the sub-healthy level to the generally healthy level. In contrast, the provinces of the sub-healthy level type further expanded, most of which were concentrated in the central and western regions. In 2020, the spatial pattern of tourism ecosystem health in China was similar to that in 2017, with only minor changes found in the type of some provinces. From the spatial distribution pattern, it can be seen that the tourism ecosystem health showed the distribution law of decreasing gradually from the eastern coastal regions to the central, western, and northeastern regions. The provinces with higher tourism ecosystem health levels, driven by both their location and policy advantages, have achieved high-quality growth in the tourism economy under the intensive mode while simultaneously optimizing the structure of the tourism industry and environmental management through advanced technological advantages. The provinces with lower tourism ecosystem health levels were mainly severely constrained by their tourism resource endowment and location conditions, which, together with their strong dependence on tourism resources, has led to bottlenecks in the development of the tourism economy.

#### 3.1.3. Dynamic evolutionary characteristics

This section used the Markov chain method to investigate the state transition of provinces in the distribution of tourism ecosystem health. It attempted to explore the long-term dynamic change trend of the differences in tourism ecosystem health between the various provinces in China. According to the type classification, the tourism ecosystem health of provinces can be discretized into four state-level types. Namely, non-healthy level (0, 0.30], sub-healthy level (0.30, 0.40], generally healthy level (0.30, 0.40] and very healthy level (0.55, 1], the completeness intervals of these four state types can be represented by k = I, II, III, and IV, respectively. The definition of an upward transfer is from a low level to a high level, and the definition of a downward transfer is from a high level to a low level.

To further understand the dynamic evolution trend of tourism ecosystem health in China, the Markov transfer matrix of tourism ecosystem health in China was obtained using Matlab R2011b software (Table 5). The value on the diagonal line represents the possibility that the province will always remain at a particular state-level type. In contrast, the value on the off-diagonal line represents the probability that the various provinces will transition from one state-level type to another. Therefore, without considering the spatial effect, the dynamic evolution of tourism ecosystem health is characterized as follows: (1) The probability value on the diagonal line in the Markov transition matrix was significantly larger than those on the non-diagonal, with a minimum value of 72.41% and a maximum value of 100.00%. Under the above conditions, tourism ecosystem health attained at least 72.41% probability in the future development process and remained at the same state-level type. The probability of maintaining stability at each state-level type was greater than the probability of the upward transfer and the downward transfer, which reflected that the club convergence of tourism ecosystem health types was highly stable, and its type transfer had a “path-dependent” and “self-locking” effect. (2) In terms of the probability value on both sides of the diagonal line, the probability of the non-healthy level type transitioning upward was 27.58%. It can be seen that the provinces with non-healthy level types were more likely to transfer upward, indicating that the tourism ecosystem health in the non-healthy level provinces was unstable and prone to state leapfrogging compared to other states. The probability of the sub-healthy level type transitioning downward was 11.88%, and the probability of the type transitioning upward was 7.92%. The probability of the downward shift of tourism ecosystem health at the sub-healthy level was more significant than the probability of the upward shift, which reflected that tourism ecosystem health still had a negative trend. The probability of the generally healthy level type transitioning downward was 27.59%, with the risk of a precipitous fall. (3) The probability values on both sides of the non-diagonal line were significantly smaller than those on both sides of the diagonal line, with a maximum value of only 0.86%, indicating that the improvement of tourism ecosystem health was a gradual process and that it was difficult to achieve leapfrogging in the short term.

**Table 5:** Markov transfer probability matrix for tourism ecosystem health types in China from 2011 to 2020.

**t/t+1**	***n***	**I**	**II**	**III**	**IV**
I	116	0.7241	0.2241	0.0431	0.0086
II	101	0.1188	0.8020	0.0594	0.0198
III	29	0.0000	0.2759	0.7241	0.0000
IV	24	0.0000	0.0000	0.0000	1.0000

The issue of spatial correlation is disregarded in the traditional Markov chain approach because it presumes that regions are independent of each other. This paper incorporated spatial lag into the traditional Markov chain analysis to investigate the neighborhood's influence and the probability that it will change among convergent groups. The results of the spatial Markov chain transition probability matrix of tourism ecosystem health in China from 2011 to 2020 are presented in Table 6.

**Table 6:** Spatial Markov probability matrix for tourism ecosystem health types in China from 2011 to 2020.

**Geospatial background**	**t/t+1**	***n***	**I**	**II**	**III**	**IV**
I	I	66	0.7273	0.1818	0.0758	0.0152
	II	22	0.0000	0.9091	0.0000	0.0909
	III	1	0.0000	1.0000	0.0000	0.0000
	IV	1	0.0000	0.0000	0.0000	1.0000
II	I	40	0.7250	0.2750	0.0000	0.0000
	II	57	0.1579	0.7895	0.0526	0.0000
	III	4	0.0000	0.5000	0.5000	0.0000
	IV	15	0.0000	0.0000	0.0000	1.0000
III	I	8	0.8750	0.1250	0.0000	0.0000
	II	16	0.0625	0.7500	0.1875	0.0000
	III	24	0.0000	0.2083	0.7917	0.0000
	IV	8	0.0000	0.0000	0.0000	1.0000
IV	I	2	0.0000	1.0000	0.0000	0.0000
	II	6	0.3333	0.6667	0.0000	0.0000
	III	0	0.0000	0.0000	0.0000	0.0000
	IV	0	0.0000	0.0000	0.0000	0.0000

The issue of spatial correlation is disregarded in the traditional Markov chain approach because it presumes that regions are independent of each other. This paper incorporated spatial lag into the traditional Markov chain analysis to investigate the neighborhood's influence and the probability that it will change among convergent groups. The results of the spatial Markov chain transition probability matrix of tourism ecosystem health in China from 2011 to 2020 are presented in Table 6.

Through comparison with Tables 5, 6, the following spatial dynamic evolution characteristics of tourism ecosystem health could be obtained after considering the geospatial background: (1) Geospatial background played a significant role in the dynamic evolution process of tourism ecosystem health. The transfer probability of tourism ecosystem health varied against the neighborhood background, implying different levels of health. It also differed from that calculated using the traditional Markov probability transfer matrix. In particular, the probability of maintaining stability at the non-healthy level type in the traditional Markov probability transfer matrix was 72.14%. In comparison, the probability of maintaining its original state-level type on different geospatial backgrounds was 72.73, 72.50, 87.50, and 0.00%, respectively, indicating a significant difference between the probability transfer of tourism ecosystem health with and without considering the geospatial background. (2) China's tourism ecosystem health exhibited a “spatial spillover” effect, with the tourism ecosystem health levels affecting each other between neighboring provinces. In general, the probability of the state-level type of province transferring downward would increase if it was adjacent to a province with a low tourism ecosystem health level. In contrast, the probability of the state-level type of province transferring upward would increase if it was adjacent to a province with a high tourism ecosystem health level. Specifically, as the state-level type of neighborhood tourism ecosystem health rose, the probability of the provinces of the non-healthy level type transitioning upward was 27.28, 27.50, 12.50, and 100.00%, showing a fluctuating upward trend; the probability of provinces of the generally healthy level type transitioning downward was 100.00, 50.00, 20.83, and 0.00%, showing a precipitous downward trend. This suggests that provinces with higher tourism ecosystem health levels have a radiation effect on their neighbors, whereas provinces with lower tourism ecosystem health levels may have an inhibition effect. Therefore, provinces with a high tourism ecosystem health level need to exploit their spillover effect actively and, through their radiation capacity, promote the health type of the surrounding provinces by leading from point to point.

### 3.2. Analysis of driving factors

In order to ensure the smoothness of the panel data time series and reduce heteroscedasticity in this paper, natural logarithms were taken as all non-percentage variables. At the same time, this paper employed both the LLC test, the Breitung test (which assumes a common unit root process), the IPS test, the Fisher-ADF test, and the Fisher-PP test (which assumes an individual unit root process) to probe the unit root properties of the study variables so that it could avoid problems such as multicollinearity and spurious regressions. According to the estimation results, every variable strongly rejected the null hypothesis that the panel had a unit root at a 1% significance level, indicating that the panel data were smooth and could be suitable for further regression analysis. This paper selected five representative quantiles for analysis, including 10, 25, 50, 75, and 90%. The Markov chain Monte Carlo (MCMC) method was adopted to estimate the driving factors of tourism ecosystem health to avoid possible endogeneity. The estimation results were presented in Columns (1) to (6) of Table 7. The changes in the quantile regression coefficients at different quantiles were displayed graphically in Figure 3. The estimated results were from stata16.0 software.

**Table 7:** The quantile regression model estimation results of driving factors of tourism ecosystem health.

**Driving Factors**	**(1)**	**(2)**	**(3)**	**(4)**	**(5)**	**(6)**
	**OLS**	**q10**	**q25**	**q50**	**q75**	**q90**
ln TIS	0.037^*^ (1.92)	0.125^***^ (3.49)	0.084^***^ (4.87)	0.059^***^ (3.25)	0.064^***^ (2.73)	0.131^**^ (3.16)
ln TIA	−0.101^***^ (−5.52)	−0.008^***^ (−0.22)	−0.010^***^ (−0.59)	−0.023^***^ (−1.24)	−0.084^***^ (−3.49)	−0.103 (−2.45)
TER	0.003^***^ (3.97)	0.003^***^ (0.85)	0.016^***^ (0.81)	−0.003 (−0.19)	−0.003 (−1.03)	−0.005 (−1.09)
ln TIC	−0.015^***^ (−4.42)	−0.020^***^ (−1.67)	−0.012 (−2.20)	−0.010^***^ (−1.70)	0.003 (−0.04)	0.007 (0.48)
ln INFO	0.016^***^ (3.69)	0.018^***^ (1.32)	0.012 (1.78)	0.013^**^ (1.89)	0.004 (0.37)	0.002^***^ (0.13)
ln TLS	0.070^***^ (12.41)	0.034^***^ (6.26)	0.033^***^ (12.31)	0.030^***^ (10.89)	0.035^***^ (9.63)	0.033^***^ (5.18)

*t* statistics in parentheses. ^*^P < 0.05, ^**^P < 0.01, and ^***^P < 0.001.

**Figure 4:**
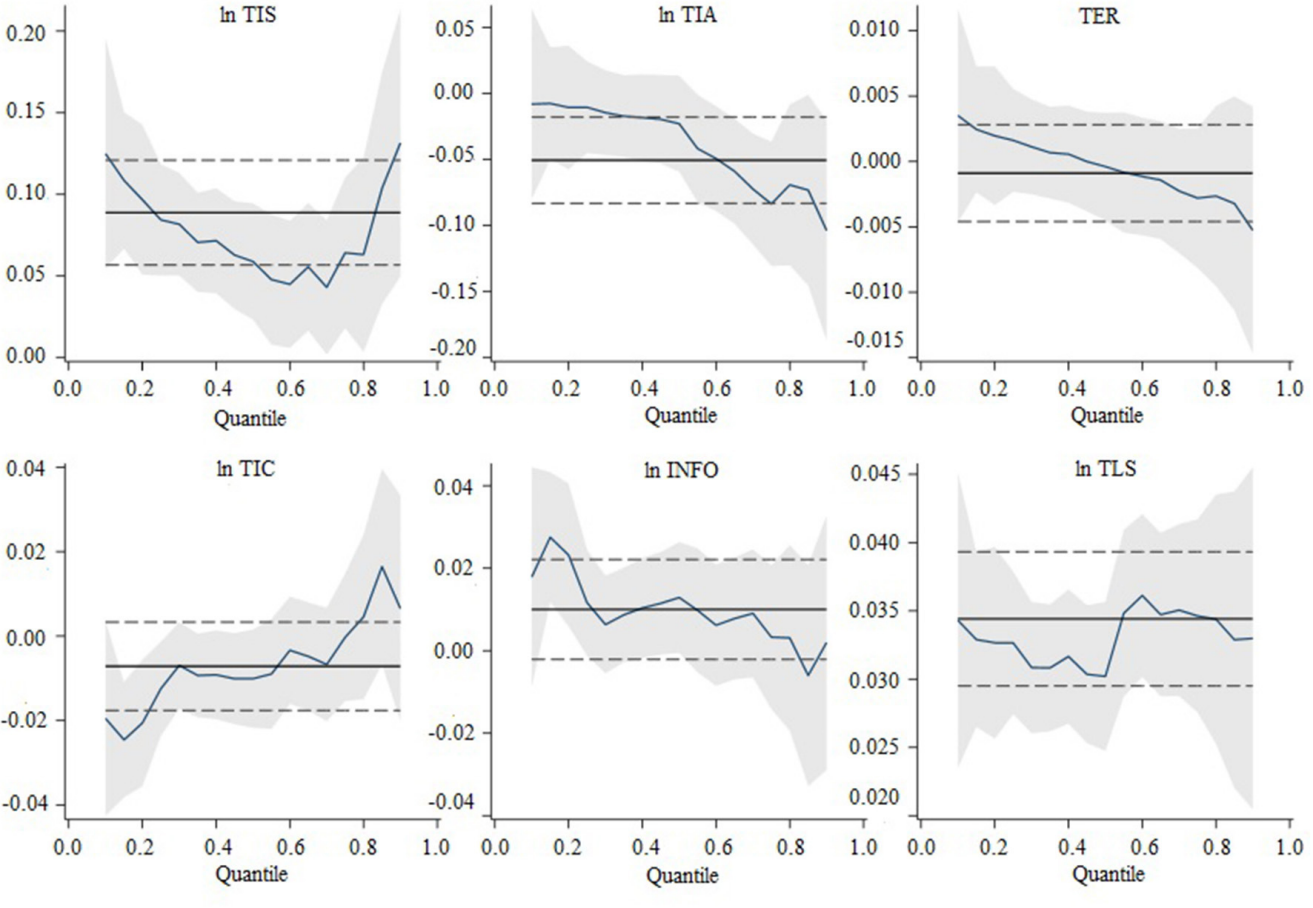
Changes in quantile regression coefficients for tourism ecosystem health.

As shown in Table 7 and Figure 4, as a whole, tourism industry agglomeration and technological innovation capacity imposed negative influences on tourism ecosystem health, and tourism industry structure, tourism environmental regulation, information technology level, and tourism land-use scale were all significant in influencing tourism ecosystem health. However, the influence coefficients of each variable at different quantiles were significantly different.

1. The results of the quantile regression of tourism industry structure (lnTIS) showed an M-shaped pattern, which was always positive and significant, indicating that the impact of tourism industry structure on tourism ecosystem health varies at different quantiles. The government should therefore transform the tourism economy and promote the integration of tourism industries to enhance tourism ecosystem health. It was important to note that the coefficient of influence of the tourism industry structure on the high quantile was relatively large. Therefore, the government should pay more attention to and strengthen the upgrading of the tourism industry structure in provinces with high tourism ecosystem health, thereby reducing duplicate construction and resource-consuming projects and increasing low-consumption, high-quality service projects, thereby promoting sustainable tourism development in these provinces.
2. At the 10, 50, 75, and 90% quantiles, the absolute value of the coefficient of tourism industry agglomeration (lnTIA) had an increasing trend, indicating that tourism industry agglomeration was the main obstacle factor for provinces with high tourism ecosystem health in China, that is, the higher the level of tourism ecosystem health, the greater the pressure of tourism industry agglomeration on the ecosystem. Consequently, our results suggested that strengthening the centralization of resource use and the use of large-scale pollution control infrastructure in provinces with high tourism ecosystem health was one of the most effective ways to increase the resilience of sustainable tourism development in such provinces.
3. Tourism environmental regulation (TER) significantly affected tourism ecosystem health at the lower quantiles, but this had no significant marginal impact as the quantiles increased. This may be because of the spatial mismatch between supply and demand in China's tourism resources and natural ecological background. The provinces with higher tourism ecosystem health types are generally more market-oriented regions, where the market itself can achieve an efficient allocation of resources through competitive mechanisms, price mechanisms, and supply and demand mechanisms. Too much macro-regulation is not conducive to forming environmental-economic systems such as green capital markets, green credit, ecological compensation, and other environmental-economic systems.
4. The influence of technological innovation capacity (lnTIC) on tourism ecosystem health was negative, with a non-significant negative effect on technological innovation capacity at the higher quartiles (75% and 90%). All other quartiles showed a significant adverse effect at the 1% level. The absolute value of the coefficient of technological innovation capacity increased as the quantile decreased, with the negative effect reaching a maximum at the 10% quantile. According to our findings, the main obstacle factor for provinces with low tourism ecosystem health in China was a lack of technological innovation capacity.
5. As the quantile changed, the value of the coefficient of information level (INFO) changed significantly, with the coefficients for the first 50% of the quantile being significantly larger than those for the second 50% of the quantile. From the coefficients, the degree of impact of information infrastructure and information technology consumption was more substantial for provinces with low tourism ecosystem health. In contrast, for provinces with high tourism ecosystem health, the effect of increasing informatization was diminished.
6. Tourism land-use scale positively affected tourism ecosystem health, with an inverted N-shaped trend. The panel quantile regression results showed that the tourism land-use scale had a significant and large positive effect on tourism ecosystem health at the high quantile. However, its positive effect decreased as the tourism land-use scale increased at this quantile. Despite the problems of waste of production factors such as land and capital and the excessive emission of pollutants in the process of the use of tourism land, the under-utilized tourism land has crowded out ecological land space. The ecosystem organization structure is under external stress. However, the resistance to external interference and self-repair functions of ecosystem services does not exceed the reasonable carrying capacity of the tourism environment.

## 4. Conclusions and discussion

Based on the DPSIR model, we systematically constructed an evaluation index system to measure China's tourism ecosystem health. Then the dynamic evolution characteristics and driving factors of tourism ecosystem health in China from 2011 to 2020 were analyzed with the entropy weight method, spatial autocorrelation analysis, Markov chain analysis, and quantile regression. The main conclusions are as follows: (1) During the ten years from 2011 to 2020, the tourism ecosystem health in China showed an M-shaped fluctuation process as a whole, with three distinct stages of rapid increase (2011–2012), slow decrease (2012–2015), and fluctuating development (2015–2020). The kernel density exhibited a right-trailing phenomenon and multiple peaks, indicating that the gap between China's tourism ecosystem health and the average has widened and that there was a specific gradient of differences. Regarding spatial differentiation, the tourism ecosystem health in China was significantly spatially correlated, characterized by clustered and contiguous development, with significant regional differences in distribution. (2) There was a “path-dependent” and “self-locking” effect on the type transfer of the tourism ecosystem health in China. Type transfer generally occurs between adjacent types in successive transfers, while the probability of cross-type transfer is small, and the probability of downward transfer is higher than upward transfer. In addition, geospatial patterns played a significant role in the dynamic evolution process of tourism ecosystem health. Specifically, the probability of the state-level type of the province transferring downward would increase if it was adjacent to a province with a low tourism ecosystem health level, while the probability of the state-level type of the province transferring upward would increase if it was adjacent to a province with a high tourism ecosystem health level. (3) The tourism ecosystem health in China was driven by a combination of factors, including tourism industry structure, tourism industry agglomeration, tourism environmental regulation, information technology level, technological innovation capacity, and tourism land-use scale. Moreover, in provinces with low tourism ecosystem health type, the dampening effect of technological innovation capacity was more significant, and the influence coefficient of the positive marginal effect of tourism environmental regulation and information technology level was larger, while in provinces with high tourism ecosystem health type, the dampening effect of tourism industry agglomeration was more significant, and the influence coefficient of the positive marginal effect of tourism industry structure and tourism land-use scale was larger.

Under the new development concept of “innovation, coordination, green, openness and sharing”, a systematic and in-depth study on the dynamic evolution of tourism ecosystem health in China and its driving factors are of great significance in promoting the sustainable development and high-quality transformation and upgrading of regional tourism. The main contributions of this paper are listed as follows: (1) The dynamic transfer process and pattern of tourism ecosystem health in each province of China from 2000 to 2015 were analyzed using the spatial Markov chain analysis, which can visually reveal the heterogeneity of the “spatial spillover” effect of tourism ecosystem health and the influence of geospatial background. (2) In contrast to the idealistic treatment model of mean regression, the panel quantile regression model emphasized the heterogeneity of driving factors in the context of various tourism ecosystem health types. Its empirical findings could more accurately reflect the actual situation. Thus, the results can provide a research methodological reference for a more comprehensive, systematic, and dynamic exploration of the driving mechanisms of tourism ecosystem health in similar areas, especially in developing countries, in the future. (3) The research scale was reduced to the provincial level, and the heterogeneity and regularity of tourism ecosystem health at the regional scale could be better explained, providing empirical support for local governments to formulate appropriate tourism ecosystem health strategies for different provinces.

The findings of this paper have important policy implications: (1) The government and tourism authorities should give due consideration to regional synergy and integrated management in tourism cooperation and development and tourism environmental protection policies as a means of reducing the constraints and impacts of spatial effects on tourism ecosystem health, reducing spatial differences in tourism ecosystem health between provinces, and achieving the goal of regional coordination and sustainable development of the tourism industry. (2) More attention should be paid to the dynamic evolution of tourism ecosystem health in different provinces, especially in provinces with a “generally healthy” neighborhood type, to avoid the risk of downward transfer resulting from the crude growth of their tourism economies. (3) The provinces with high tourism ecosystem health must make efforts to accelerate the process of allocating tourism industry elements, continuously promote structural reform on the supply side of tourism, guide tourism development and transformation and upgrading with the concept of ecological priority and green development, and actively cultivate new low-carbon and green tourism industries. Meanwhile, actions must be taken to initiate the allocation of land resources in ways that are compatible with the direction that the tourism industry is going as well as through intensive utilization for sustainable development. For provinces with low tourism ecosystem health, the research and development of tourism pollution treatment and prevention technologies should be focused on further improving tourism ecosystem health. Additionally, regional tourism ecosystem health policies should be continuously improved. Tourism ecosystem health should be promoted through new media platforms to encourage green travel and low-carbon consumption among tourists.

## Data availability statement

The original contributions presented in the study are included in the article/supplementary material, further inquiries can be directed to the corresponding author.

## Author contributions

FL: conceptualization, software, data curation, and writing-original draft preparation. HR: methodology and writing-reviewing. XZ: editing and visualization. All authors contributed to the article and approved the submitted version.
